# Pareto Optimization Identifies Diverse Set of Phosphorylation Signatures Predicting Response to Treatment with Dasatinib

**DOI:** 10.1371/journal.pone.0128542

**Published:** 2015-06-17

**Authors:** Martin Klammer, J. Nikolaj Dybowski, Daniel Hoffmann, Christoph Schaab

**Affiliations:** 1 Evotec (München) GmbH, Dept. of Bioinformatics, Am Klopferspitz 19a, 82152 Martinsried, Germany; 2 Center for Medical Biotechnology, University of Duisburg-Essen, Universitätsstrasse 1-4, 45141 Essen, Germany; 3 Max-Plack Institute for Biochemistry, Am Klopferspitz 18, 82152 Martinsried, Germany; University of Westminster, UNITED KINGDOM

## Abstract

Multivariate biomarkers that can predict the effectiveness of targeted therapy in individual patients are highly desired. Previous biomarker discovery studies have largely focused on the identification of single biomarker signatures, aimed at maximizing prediction accuracy. Here, we present a different approach that identifies multiple biomarkers by simultaneously optimizing their predictive power, number of features, and proximity to the drug target in a protein-protein interaction network. To this end, we incorporated NSGA-II, a fast and elitist multi-objective optimization algorithm that is based on the principle of Pareto optimality, into the biomarker discovery workflow. The method was applied to quantitative phosphoproteome data of 19 non-small cell lung cancer (NSCLC) cell lines from a previous biomarker study. The algorithm successfully identified a total of 77 candidate biomarker signatures predicting response to treatment with dasatinib. Through filtering and similarity clustering, this set was trimmed to four final biomarker signatures, which then were validated on an independent set of breast cancer cell lines. All four candidates reached the same good prediction accuracy (83%) as the originally published biomarker. Although the newly discovered signatures were diverse in their composition and in their size, the central protein of the originally published signature — integrin β4 (ITGB4) — was also present in all four Pareto signatures, confirming its pivotal role in predicting dasatinib response in NSCLC cell lines. In summary, the method presented here allows for a robust and simultaneous identification of multiple multivariate biomarkers that are optimized for prediction performance, size, and relevance.

## Introduction

Targeted drugs, such as kinase inhibitors, are extensively studied as promising agents either alone or in combination with other agents for treating cancer. Unfortunately, only subsets of patients usually respond to targeted therapeutic interventions. Tests that can predict whether patients will benefit from these therapies are therefore desired companions of targeted drugs. Many, if not all response prediction tests currently used in clinical practice are based on markers directly linked to the disease-relevant drug target. However, singleton markers measuring the expression or mutation status of a drug target may often not be sufficient to predict response. For example, it has recently been shown that the success of predicting how melanomas respond to targeted therapies by genotyping alone may be limited [1].

Therefore, several studies have focused on identifying molecular signatures comprising multiple markers for response prediction. Predominantly, these signatures were identified using transcriptomics data (for example [2, 3]). In recent years, advances in sample processing, mass spectrometry, and computer algorithms for the analyses of proteomics data have enabled the application of mass spectrometry-based proteomics in order to monitor phosphorylation events in a global and unbiased manner [4–6]. These methods have become sufficiently sensitive and robust to identify and quantify thousands of phosphorylation sites in a single experiment. Phosphorylation events play an important role in transduction of signals caused by external stimuli from the cell membrane to the nucleus. Aberrations of these signal transduction pathways are important for the understanding of the molecular mechanism of various diseases, such as cancer, inflammation, and diabetes [7, 8]. At the same time, many of the targeted therapies interfere with these kinase signaling pathways. Consequently, multivariate markers based on the phosphorylation status of certain sets of proteins—here referred to as phospho-signatures—are assumed to reliably predict the clinical response. This has been demonstrated in two recent studies, where phosphoproteomics data was used to identify predictive multivariate markers for the multi-kinase inhibitor dasatinib [9] and the FLT3 inhibitor quizartinib [10].

Previous studies have focused on the identification of *one* single multivariate marker signature that was optimized for prediction accuracy. Here, we investigate a method that allows for the incorporation of additional objectives that are optimized simultaneously, and enables the identification of *several* predictive marker signatures. Such objectives can, for instance, be related to the annotations of protein markers (e.g. localization, function), to technical properties (e.g. size of the signature), or to network information (e.g. proximity of markers to drug target). Recently, it has been shown in a study analyzing 44 drug sensitivity prediction algorithms that the impact of including annotated biological information is stronger than the impact of the method category (e.g. support vector machine, random forest, etc.) or the method for missing data handling [11]. More specifically, it has also been demonstrated that adding network information can improve prediction accuracy or at least improve the robustness of feature selection (e.g. [12]). These methods have in common that the network information is factored in by modifying the objective function (e.g. network-based support vector machines [13]) or the rank order for filter-based feature selection (e.g. NetRank [14, 15]). However, instead of optimizing a combined objective function, we choose to optimize multiple objectives in parallel using principles of multi-objective or, specifically, Pareto optimization [16]. Multi-objective optimization methods return a set of optima, the so-called Pareto front, instead of a single optimum solution. In case of selection of predictive biomarkers, these solutions differ in their composition of selected features and in the degree to which different objectives are optimized. If necessary to limit the number of marker candidates, the researcher can apply *post hoc* weighting of objectives.

In biomedical research, Pareto optimization has been mainly applied to design of small molecules [17] and peptide sequences [18]. More recently however, it has also been applied to selection of features. For example, Rajapakse and Mundra optimized features for multi-class classification by decomposing the over-all objective to multiple objectives for each pair of classes [19]. Xue et al. complemented the objective of classification accuracy with minimizing the size of the signature [20]. Here, we generalize the idea of applying Pareto optimization to the problem of selecting predictive marker signatures by optimizing not only the prediction accuracy and the size of the signature, but also the biological relevance of the selected features. We define the biological relevance by the proximity of features to the respective drug target as derived from protein-protein interaction networks. In principle, all obtained solutions on the Pareto front can be evaluated and tested in validation experiments. However, since in practice the Pareto front consists of several dozens of solutions, we propose to cluster these solutions in feature space and investigate a much smaller number of cluster centroids. We apply the proposed method to the identification of multivariate phosphorylation signatures that predict response to dasatinib in non-small cell lung cancer and breast cancer cell lines using the phosphoproteomics data generated by Klammer et al. [9].

## Results and Discussion

The main goal of response prediction biomarker studies is the identification of molecular signatures that separate the group of responders from the group of non-responders well and thus enable an accurate prediction of drug response. However, there are further qualities that characterize a successful biomarker. For example, a marker should consist of a manageable number of features (i.e. genes or proteins) in order to allow testing through methods applied in clinical routine such as quantitative PCR or ELISA. Furthermore, the features should be biologically relevant, for instance, by being connected to the drug’s target or mechanism of action. In the proposed Pareto biomarker workflow, these three objectives—separation, signature size and relevance—are optimized in parallel (for definitions of objectives see section Pareto objective functions in Materials and Methods).

### Pareto biomarker workflow

To this end, a multi-objective optimization algorithm (MOA) was incorporated into our established biomarker discovery workflow [9], allowing the simultaneous optimization of all three objectives. Most MOAs employ the principle of Pareto optimality, which aims at detecting solutions that are not dominated by other solutions. At any given iteration, non-dominated solutions are defined such that there exist no other solutions that have a better or equal score in *all* objectives and a strictly better score in *at least* one objective. All non-dominated solutions (Pareto points) together form the Pareto front (see also S1 Fig), which is optimized during each iteration. Of the many MOA algorithms available (e.g. PAES [21], PESA [22], SPEA2 [23], NSGA-II [24] or SMS-EMOA [25]), we found the NSGA-II algorithm [24] most suitable for our Pareto biomarker workflow, as it shows fast convergence, is efficient and well tested [26, 27].

In a previous study, we used quantitative mass-spectrometry to globally profile the basal phosphoproteome of a panel of 19 non-small cell lung cancer (NSCLC) cell lines [9]. The effect of the kinase inhibitor dasatinib on cellular growth was tested against the same panel. Using the phosphoproteome data, we identified a phosphorylation signature consisting of 12 phosphorylation sites on 9 different proteins. The signature accurately predicted response to treatment with dasatinib in the NSCLC cell lines used for training and in an independent validation panel of breast cancer cell lines.

Here, we investigated whether the Pareto biomarker workflow could confirm the original signature and/or identify additional multivariate predictive phosphorylation signatures when applied to the same data set. In particular, these signatures should not only maximize class separation, but also the two additional objectives *signature size* and *relevance*. We hypothesize that if a marker protein is closely related to the drug target (e.g. through interaction), this protein is more relevant in the sense that a signature consisting of such proteins will lead to more robust predictions when applied to diverse sets of samples. Although this might not always be the case, we think that this is a good assumption on average. Since this is only one out of three objectives to be optimized, signatures that are not connected to the drug’s target may still be identified and are not discarded. More specifically, we define the relevance score of a signature as the average distance of the signature’s proteins to dasatinib’s main target in solid tumors, the Src kinase (SRC), as it has been shown that dasatinib inhibits migration and invasion of various solid tumors through inhibition of SRC [28–30]. All three scores are defined such that smaller values are better. Thus, all three objectives are to be minimized (see Materials and Methods for details).

**Fig 1:**
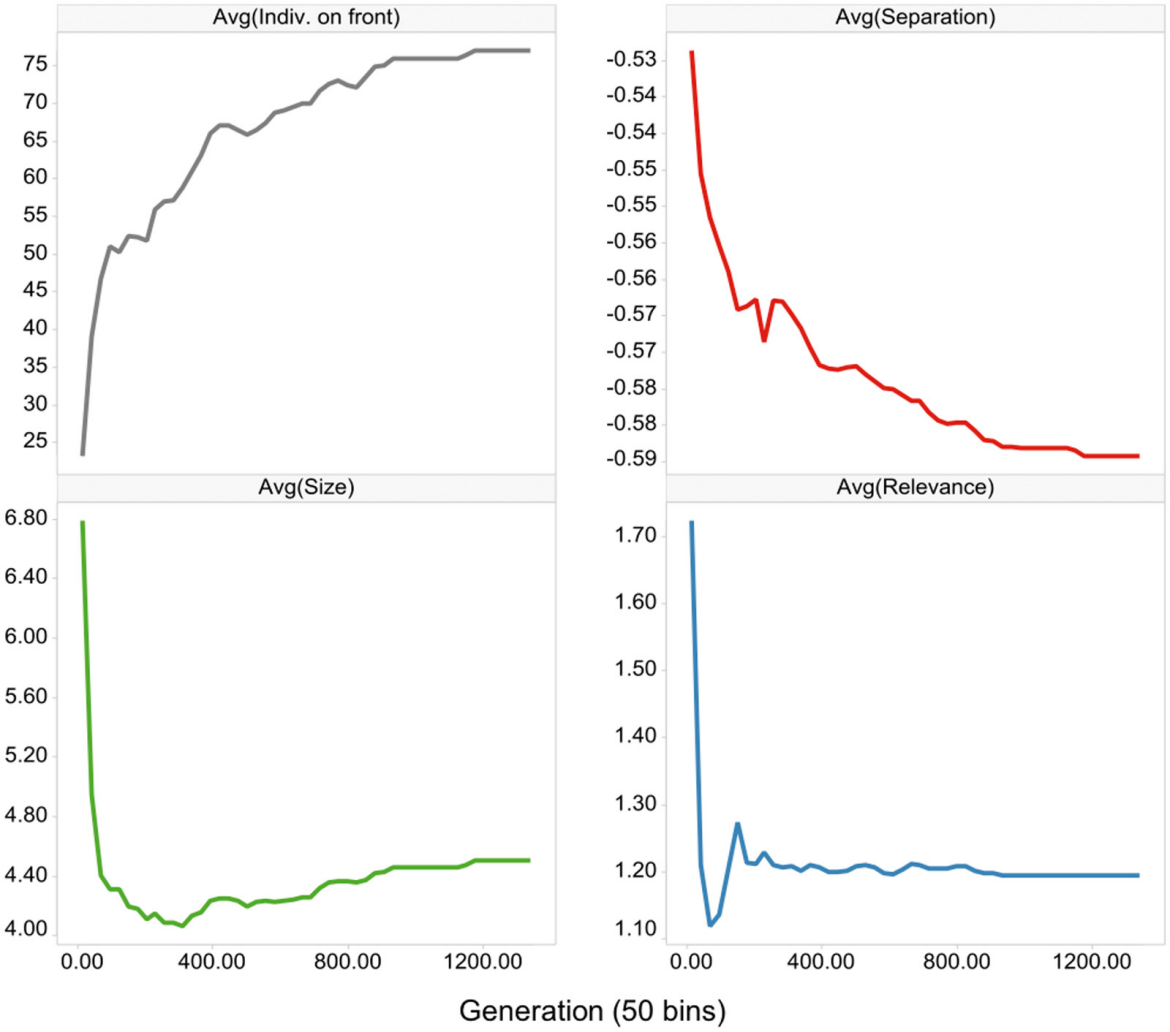
Evolution of the number of individuals (solutions) on the Pareto front and for the three objectives (separation, size and relevance), as generated by the NSGA-II algorithm [24]. The objectives are averaged across the solutions on the Pareto front. The number of generations is binned and the average of each bin is displayed on the y-axis.

From the 4,457 phosphorylation sites quantified in at least 2/3 of the samples in each class (responders and non-responders), we selected the 100 sites that discriminated best between responders and non-responders according to the MeanRank test [31], while ensuring that the mean difference between the two groups was at least 4-fold and only one phosphosite per protein was taken. This pre-selection was performed to reduce the complexity of the subsequent Pareto optimization. The algorithm terminated after 1353 generations, at which point the results on the first Pareto front had not changed for 200 generations (Fig 1). While the number of solutions on the Pareto front constantly increased, the three objectives (i.e. separation, size and relevance) were minimized with respect to Pareto optimality. As can be deduced from the graphs of the three objectives, the size and relevance criteria are rather easy to optimize, as they exhibit a steep decline at the beginning of the optimization process and reach the global minimum early on. Optimization of the separation criterion took longer and its decrease in the later stages was accompanied with an increase of the size objective, while the relevance criterion remained stable. In essence, small signatures that have a short distance to the drug target in the STRING protein-protein interaction (PPI) are readily discovered. It is, however, harder to find those that additionally separate the groups well and, essentially, good separation comes at the cost of larger signatures.

After termination, 77 solutions were located on the Pareto front. Solutions with a separation score ≥ −0.6 were removed (*N* = 24), as the Pareto approach also found very small and biologically relevant solutions with poor separation. This is an inherent feature of Pareto optimization, and removing undesired solutions is common practice (see e.g. [32]). The remaining 53 solutions contained 35 different phosphorylation sites. One site, S1148 on integrin β4 (ITGB4), was part of all but three solutions.

**Fig 2:**
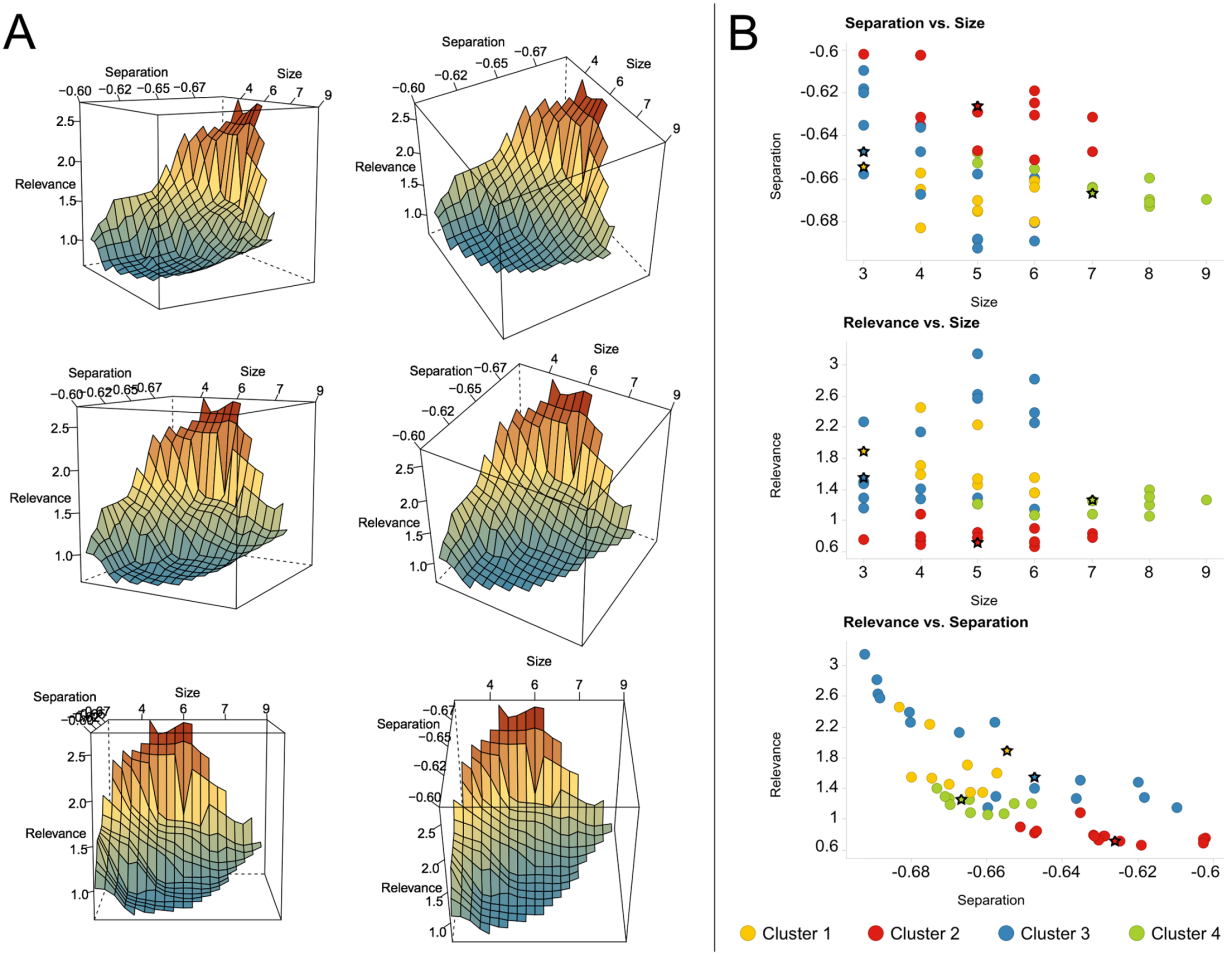
3D plots of the Pareto front (A) and 2D projections (B). (A) The different panels are views of the Pareto front when rotated around the relevance-axis (with two different viewing angles in both columns). Coloring indicates relevance score, from blue (low) to red (high). Since better solutions are smaller in all three dimensions, the optimal point is the origin in the lower background, i.e. the only hidden vertex in the plots. (B) 2D projections of the solutions on the Pareto front. Solutions are colored according to their assignment to four clusters. Stars mark the solutions closest to the respective cluster centroid that were selected as final Pareto signatures.

Fig 2A shows a series of three-dimensional plots of the Pareto front. The front has the shape of a stretched canvas attracted by the origin, which represents an ideal but infeasible point. Fig 2B depicts 2D projections of the 53 Pareto front solutions in objective space. The top panel, relating size and separation, shows that smaller signatures lead to less pronounced separation and illustrates our initial motivation for identifying multivariate markers. Therefore, the lower left corner in the plot, where ideal solutions for the two respective objectives are expected, is not populated. However, there are also no large signatures in the area of the best-separating solutions (< −0.68). This is due to the third objective, the relevance criterion, as it becomes harder to identify features that all interact directly or indirectly with the target. As mentioned before, the task of finding small and biologically relevant solutions is achieved more easily, as can be seen in the center panel of Fig 2B. Solutions are found in the lower left area, but not in the lower right. The bottom panel of Fig 2B depicts the relationship between separation and relevance. This projection of the Pareto front has a curved shape, revealing the compromise between good separation and biologically meaningful features, as not all well-discriminating phosphosites are also related to the drug target.

### Pareto signatures

**Fig 3:**
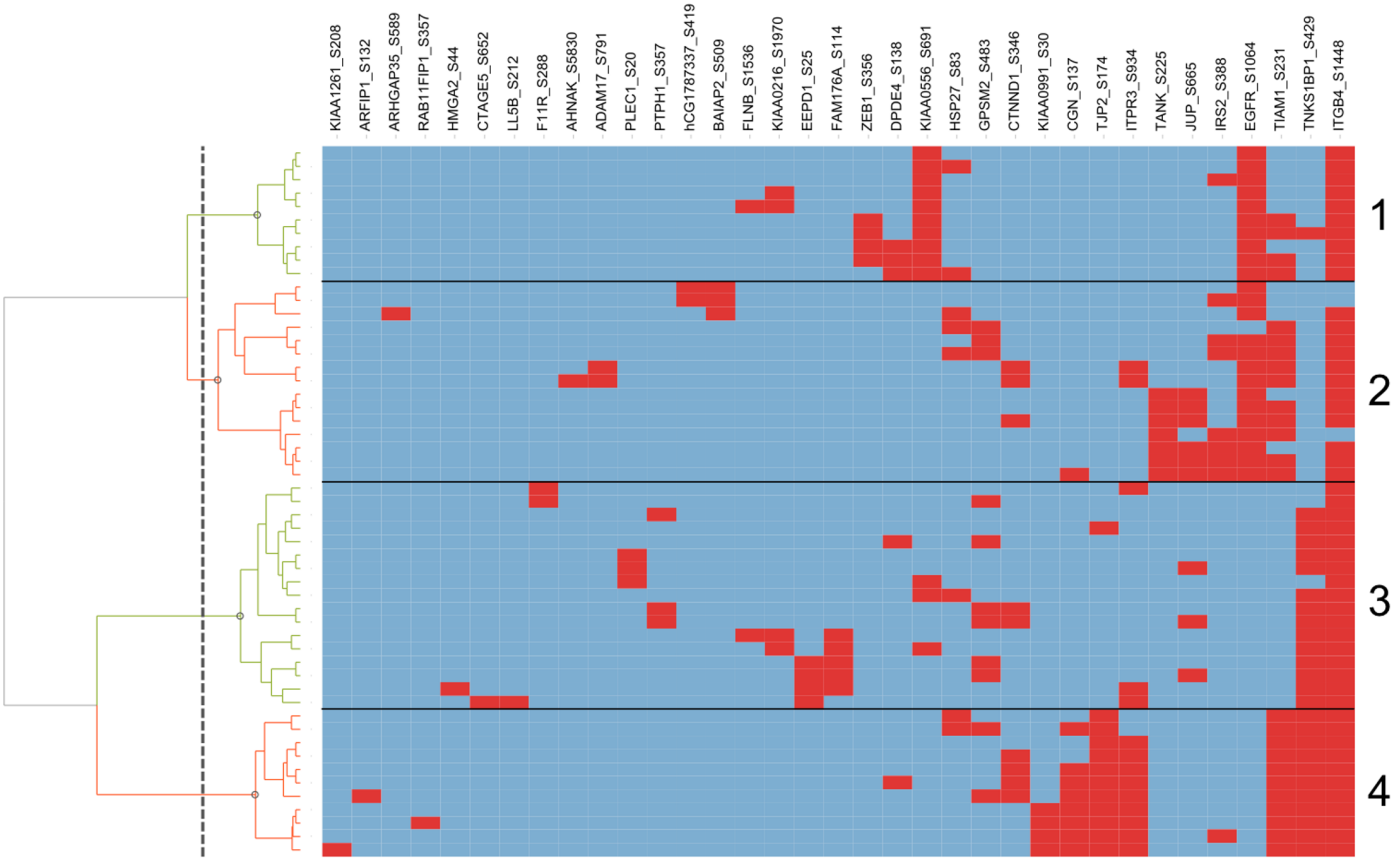
Hierarchical clustering of the 53 accepted solutions on the Pareto front in feature space. In each row, red areas represent features (phosphosites) that are part of the corresponding solution. The solutions were subdivided into four clusters according to the row dendogram on the left. Cluster numbers are indicated on the right.

Each of the identified solutions on the Pareto front is optimal in the sense that none of them are dominated by any other solution. Therefore, each solution could be evaluated individually. Here we took another approach and investigated whether solutions can be reduced by clustering according to their similarity while retaining discriminatory power. To this end, we hierarchically clustered the solution in features space using the Ward method and obtained four major clusters (see Fig 3). For each of these clusters, the feature with the smallest Euclidean distance to the respective cluster centroid was selected as so-called *Pareto signature* for further analysis (see Fig 2B).

**Fig 4:**
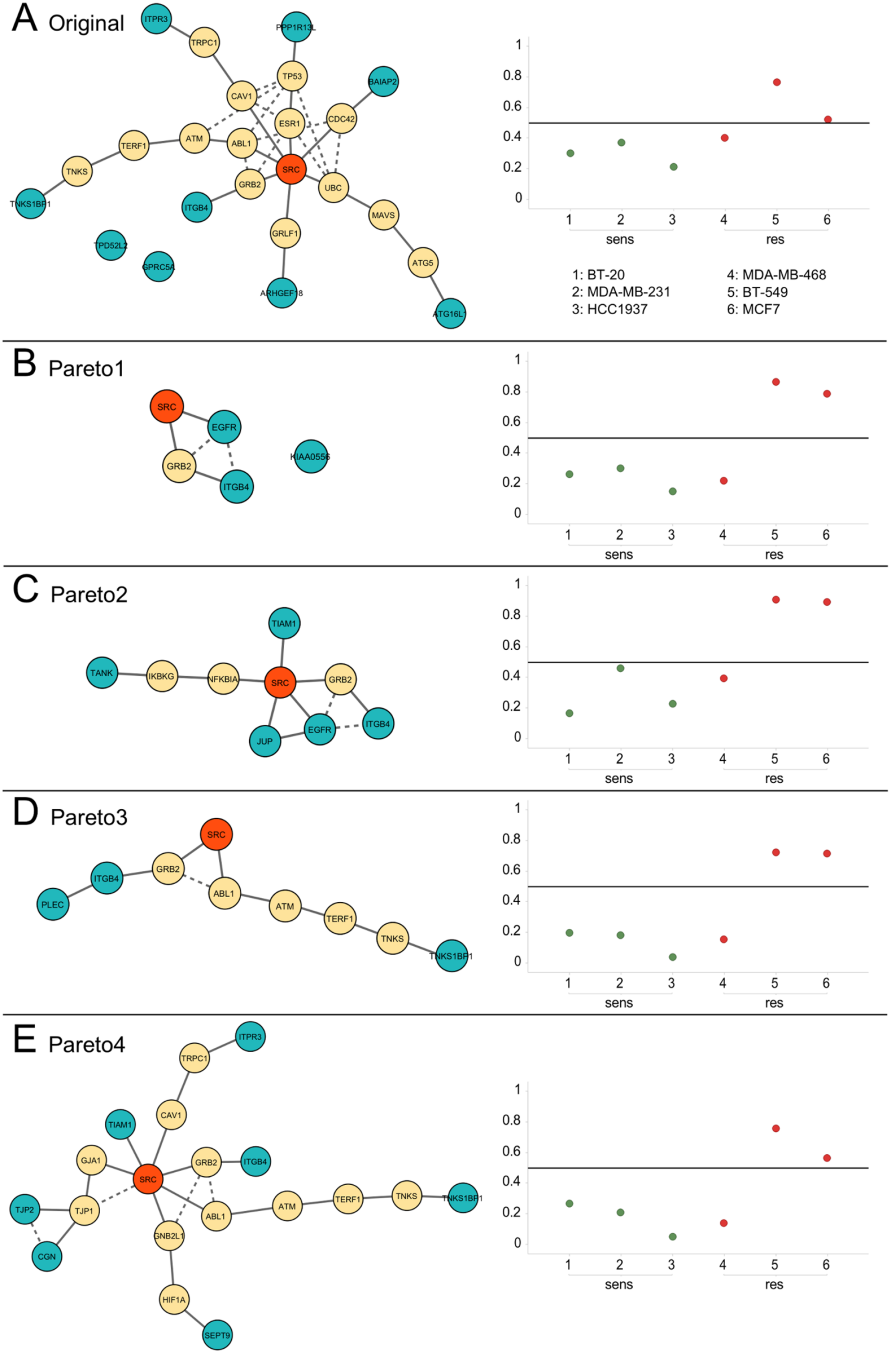
Network of the original (A) and the four Pareto signatures (B-E) are shown in the left column along with the prediction results for the breast cancer validation set (right column). Blue nodes in the network are signature proteins, solid lines represent the shortest path to SRC, dashed lines indicate additional high-confidence interactions in the network. Sensitive (1–3) and resistant (4–6) breast cancer cell lines were considered to be predicted correctly if they received a probability (y-axis) of less than 0.5, or more than 0.5, respectively.

In order to compare the original 12-phosphosite signature with the Pareto signatures, we calculated its objective values: *size* = 12, separation = −0.60, relevance = 1.63 (see also Table 1). Note, that the original signature was optimized with respect to prediction accuracy only, and the feature selection method did not explicitly optimize the separation criterion as defined here (see Materials and Methods). Fig 4A shows the PPI network of the original marker, where solid lines indicate the shortest path from each signature phosphoprotein (blue) to SRC (red), which is dasatinib’s main target in solid tumors. The phosphorylation sites of the signature are listed in Table 2 (referred to as original signature). Some of the signature proteins ITGB4, ARHGEF18 and BAIAP2 are closely related to SRC, while others (e.g. ATG16L1 and TNK1SBP1) have larger distances in the PPI network. TPD52L2 and GPRC5A have no connection to SRC at all. In the original publication [9], the selected features were used to train a support vector machine (SVM) with linear kernel. The signature and the corresponding predictor were then validated by application to six independent breast cancer cell lines, which had not been used for feature selection or SVM training. In the case of the original signature, five out of six cell lines were predicted correctly with an average probability distance to the hyperplane of 0.13 (calculated as $\frac{1}{N}\sum_{i=1}^{N}(0.5-p_{i})c_{i}$, where *N* is the number of tested cell lines, *p*_*i*_ the prediction probability for the cell line to be resistant, and *c*_*i*_ the actual class of the cell line (sensitive = 1, resistant = −1)).

**Table 1:** Objective scores (smaller are better), prediction accuracy and average probability distance for the validation data (larger are better).

Signature	Size	Separation	Relevance	Validation accuracy	Validation distance
Original	12	-0.60	1.63	5/6	0.13
Pareto1	3	-0.65	1.88	5/6	0.19
Pareto2	5	-0.63	0.72	5/6	0.22
Pareto3	3	-0.65	1.55	5/6	0.19
Pareto4	7	-0.67	1.26	5/6	0.15

**Table 2:** Phosphorylation sites of the final signatures. Sites/proteins in bold are part of the original signature [9].

Signature	Accession	Gene name	Site
Original	P16144-2	ITGB4	S1448
	Q9UQB8-5	BAIAP2	S509
	P16144-2	TGB4	S1387
	P16144-2	TGB4	T1385
	P16144-2	TGB4	S1069
	A8K556	GPCR5A	S345
	Q14573	ITPR3	S916
	Q9C0C2	TNKS1BP1	S429
	Q6ZSZ5	ARHGEF18	S1101
	Q8WUF5	PPP1R13L	S102
	Q676U5	APG16L	S269
	O43399-2	TPD52L2	S141
Pareto1	**P16144-2**	**ITGB4**	S**1448**
	A9CB80	EGFR	S1064
	O60303	KIAA0556	S691
Pareto2	B2R7S3	TANK	S225
	**P16144-2**	**ITGB4**	**S1448**
	A9CB80	EGFR	S1064
	Q13009	TIAM1	S231
	P14923	JUP	S665
Pareto3	**P16144-2**	**ITGB4**	**S1448**
	Q15149-4	PLEC1	S20
	**Q9C0C2**	**TNKS1BP1**	**S429**
Pareto4	**P16144-2**	**ITGB4**	**S1448**
	**Q9C0C2**	**TNKS1BP1**	**S429**
	Q9UDY2	TJP2	S174
	B9EK46	CGN	S137
	Q9UHD8	SEPT9	S30
	Q13009	TIAM1	S231
	**Q14573**	**ITPR3**	S934

The first Pareto signature (Pareto1, Fig 4B) contains only three phosphoproteins—ITGB4 (integrin β4) S1448, EGFR (epidermal growth factor receptor) S1064 and KIAA0556 (uncharacterized protein KIAA0556) S691—for details see Table 2. While the separation and size objective scores are better than those of the original signature (see Table 1), the relevance score is slightly worse, which is due to the uncharacterized protein KIAA0556 that lacks functional annotation and therefore has no connection with SRC. The prediction accuracy on the validation set is comparable to that of the original signature, however, the average probability distance to the SVM hyperplane is slightly higher and thus better. Phosphorylation site S1448 on ITGB4 is one of the best separators in the data set and is also part of the original signature. ITGB4 is linked to the Src-Fak pathway [33] and is associated with poor patient prognosis [34–36]. The EGF receptor can be phosphorylated by the Src kinase [37], and is therefore directly linked to SRC in the protein-protein interaction network (STRING confidence score of 0.999).

The signature Pareto2 (Fig 4C), contains the same phosphosites on ITGB4 and EGFR, and additionally TANK (TRAF family member-associated NF-kappa-B activator) S225, TIAM1 (T-lymphoma invasion and metastasis-inducing protein 1) S231 and JUP (Junction plakoglobin) S665—see also Table 2. This signature has a particularly good relevance score (cf. Table 1), which is also visible in the PPI network, where 4 out of 5 proteins are closely connected to SRC. The performance on the validation data is comparable to that of Pareto1.

The third Pareto signature (Pareto3, Fig 4D) is another small signature containing sites S1448 on ITGB4, S20 on PLEC1 (Plectin) and S429 on TNKS1BP1 (182 kDa tankyrase-1-binding protein). S429 on TNKS1BP1 is also part of the original signature, together with S1448 on ITGB4. TNKS1BP1 has a rather large distance to SRC, leading to a mediocre relevance score. The other scores are identical to those of Pareto1, the second 3-phosphosite signature (cf. Table 1).

Finally, the largest Pareto signature (Pareto4, Fig 4E), contains ITGB4 S1448, TNK1SBP1 S429, TJP2 (Tight junction protein ZO-2) S174, CGN (Cingulin) S137, SEPT9 (Septin-9) S30, TIAM1 S231 and ITPR3 (Inositol 1,4,5-trisphosphate receptor type 3) S934. Again, the sites on ITGB4 and TNKS1BP1 are those that are part of the original signature. ITPR3 appears in the original signature with a different phosphosite (S916).

Taken together, Pareto markers are consistently smaller than the original marker, while three of four also have better separation and relevance scores. The prediction accuracy on the validation set is identical for all investigated signatures, however, the average probability distance to the separating SVM hyperplane is slightly higher for the Pareto signatures, suggesting that the Pareto signatures are more robust when being applied to other classes of related tumor cell lines.

## Conclusions

We and others have previously shown that the identification of response prediction markers from phosphoproteomics experiments in pre-clinical or clinical settings is possible [9, 10, 38]. These studies sought to identify single signatures of phosphorylation sites maximizing the separation on the data used for training. Here, we investigated the idea of integrating additional objectives, such as the relevance with respect to the drug target or the size of a signature, into the feature selection process. We applied the multi-objective genetic algorithm NSGA-II [24] to the identification of Pareto-optimal solutions for the prediction of response of NSCLC cell lines to treatment with dasatinib. Beside separability, we used the proximity of markers to the main drug target—the Src kinase—and the size of the signature as objectives for optimization.

In total, the algorithm identifies 77 Pareto-optimal solutions, i.e. solutions that are not dominated by any other solution. Each solution corresponds to a phosphorylation signature that can be used for response prediction. 53 of them had a sufficiently good separation score and were considered in the following analysis. Clustering of these solutions in feature-space revealed four groups of solutions with similar sets of phosphorylation sites. We used the solution closest to the centroid of each cluster as representatives of the four Pareto signatures. All four signatures predicted the response of six breast cancer cell lines that were not used for training with good accuracy (83%). The same accuracy was also reached by the original 12-marker signature identified in Klammer et al. [9]. Since NSCLC and breast cancer are often caused by aberrations of different signaling pathways, predictive signatures may be expected to differ in NSCLC and breast cancer. The fact that the four signatures identified here all have a high accuracy in NSCLC and breast cancer demonstrates their reliability and, particularly, their robustness.

The phosphorylation site S1448 on ITGB4 (Uniprot accession: P16144-2; or, equivalently, S1518 in the canonical sequence P16144-1) is the central feature in all four Pareto signatures. This is in accordance with the results from Klammer et al. [9], where the same site was ranked first in the robust feature selection approach. Furthermore, ITGB4 is closely linked to dasatinib’s main target SRC via the adapter protein GRB2. Thus, the Pareto marker approach is consistent with the outstanding role of ITGB4 in predicting dasatinib response in cancer cells.

Although it was not in the scope of the study to perform a comprehensive comparison between many different feature selection approaches applied to various diverse data sets, we compared the Pareto optimization approach to the biomarker discovery workflow established in the previous publication [9]. The forward feature selection method used there yielded a larger signature containing 12 phosphorylation markers, while the incorporation of signature size as additional objective in the genetic algorithm approach led to relatively small signatures (between 3 and 7 phosphorylation sites) in the present study. Similarly, optimizing for biological relevance led to signatures containing many proteins that are directly or indirectly linked to the Src kinase. More specifically, we used the proximity of the markers to the drug target SRC as determined in the STRING PPI network as proxy for the biological relevance of the markers. Despite advances in the determination of protein-protein interactions [39], PPI networks are still notoriously noisy. We therefore took only interactions into account that had high confidence scores (≥ 0.9). Moreover, since the PPI network is used only to complement the phosphoproteomics data, the effect of false positives on the selection of features will be limited as long as the corresponding phosphorylations do not exhibit predictive differential regulations.

The four Pareto signatures are characterized by properties that correspond to the objectives used for optimization. Signature 1 and 3 are relatively small with only three phosphorylation sites each. On the other hand, signature 4 is larger (7 sites), but has the best separation. Finally, signature 2 shows the best relevance score, meaning that its marker proteins are interacting with the drug target SRC either directly or through intermediate proteins. Surprisingly, while its separation on the training data is the smallest of all four signatures, it yields the highest separation on the breast cancer cell lines that were used for validation. This hints at the importance of incorporating network information in general and the relationship to the drug target in particular for the identification of robust predictive marker, which can be applied to diverse sets of samples, e.g. breast cancer instead of NSCLC cell lines.

Of course, Pareto optimization is not the only possibility to incorporate network information or other kind of additional information into the identification of predictive markers. For example, Deng, Geng and Ali applied a Bayesian Network model to integrate mass spectrometry and microarray data [40]. The top-performing team in the NCI-DREAM drug-sensitivity prediction challenge applied a nonlinear, probabilistic regression model [11]. These approaches try to include the different data sources into a unified model, whereas here we model the different data sources as separate objectives. Although the approaches are based on different principles, the aim to integrate additional data sources is the same. Indeed, it would be very interesting to combine these approaches—for example, by using a Bayesian network as basis for additional objectives.

We optimized the selected features with respect to the objectives separation, size, and relevance. Naturally, the proposed method can be applied to other objectives. For example, it may be sensible to include the detectability of marker phosphorylations in immunoassays, the localization of the marker proteins (e.g. cell membrane, nucleus, or cytosol), or the extend of knowledge about the proteins (e.g. number of PubMed abstracts).

Aside from the possibility of incorporating multiple objectives into the selection of the biomarker signatures, an even more important advantage of the approach presented here is the identification of several independent signatures instead of only one. These signatures can be evaluated post-hoc using additional criteria before a final signature or a set of a few signatures is selected for further validation experiments.

In summary, we demonstrated the power of Pareto optimization when applied to identification of predictive phosphorylation signatures. Naturally, the approach is by no means restricted to this kind of data and could equally well be applied to other high-dimensional data such as transcriptomics, genomics, or metabolomics data. Besides optimizing the separation between two classes, the method allows the consideration of additional objectives. In particular, we showed that the relation of the marker proteins to the drug target in a protein-protein interaction network can improve the robustness of the prediction when applied to new samples.

## Materials and Methods

### Data

The training data comprising the class-I phosphorylation site ratios of 19 NSCLC cell lines relative to a SuperSILAC spike-in were obtained from Supplemental Table 3 of Klammer et al. [9]. The validation data for 6 breast cancer cell lines were taken from Supplemental Table 4 of the same source. Detailed information about the generation of both datasets is provided in the main article of Klammer et al. [9]. In brief, the dataset contains more than 25,000 class-I phosphorylation sites (i.e. sites with high localization confidence), contaminant and reverse database hits were removed, and the normalized ratios (cell line versus SuperSILAC) were log10-transformed.

### Pareto objective functions

Three objectives were considered: signature size, separation and relevance.

#### Signature size

This objective score is defined by the number of phosphosites in a given signature. The score is to be minimized.

#### Separation

This objective focuses on the generalization of the marker. Not only should a good marker separate the training data, but also unseen data. Thus, an inner leave-one-out cross validation was performed by employing a support vector machine with linear kernel and cost parameter *C* = 1. For each test sample, its distance of to the SVM hyperplane was computed and the posterior class probability was calculated from a sigmoid model $p_{i}=\frac{1}{1+\text{exp}(Af_{i}+B)}$, where *p*_*i*_ is the probability of the sample being resistant and *f*_*i*_ the SVM output of the respective training data [41]. Parameter *A* was determined by optimizing a regularized maximum likelihood problem (see [41] for details); parameter *B* was fixed to 0, so that points on the separating hyperplane are assigned a probability of 0.5. The separation objective corresponds to minimization of the negative minimal probability distance $-{\text{min}}_{i}(c_{i}(\frac{1}{2}-p_{i})+\frac{1}{2})$, where *c*_*i*_ is the actual class of the cell line (sensitive = 1, resistant = −1).

#### Relevance

This objective deals with the relevance of a signature with respect to the drug target. Here, the mean distance of the signature’s proteins to the target of the investigated drug in a protein-protein interaction network (STRING) was calculated. To this end, we calculated an adjacency matrix using all interactions with a interaction confidence larger than 0.9 (STRING version 9.05 [42]). The remaining edges with interaction confidence scores *s* ranging from 0.9 to 0.999 were transformed into a penalty score using the equation $\rho_{i}=\frac{1}{-{\text{log}}_{10}(1-s_{i})}$, ranging from 0.33 to 1. The function was chosen to get more pronounced differences between higher and lower confidence scores. Subsequently, the shortest path between each protein in the signature and the drug target based on the penalties *ρ* was calculated with the Dijkstra algorithm [43] and the mean distance of all signature proteins was used as objective score.

### Pareto optimization

For the detection of the Pareto front, we applied the NSGA-II algorithm. NSGA-II is a fast, elitist multi-objective genetic algorithm [24] that employs the principle of Pareto optimality. In brief, the algorithm works as follows: First, a random parent population *P*_0_ was generated that consists of *N* = 200 chromosomes. The representation of the chromosome is binary, i.e. a feature that is part of the signature is represented by 1, a feature that is not part of the signature by 0. The chromosomes were randomly initialized with 10% of features set to 1. Next, a fitness value was assigned to each chromosome, which represents the Pareto front the individual was located on. A fitness value of 1 stands for a solution on the first Pareto front, 2 for a solution on the second Pareto front, and so forth.

Subsequently, 5-way-tournament selection, single-point crossover (*p* = 0.8) and bit flip mutation (*p* = 0.02) were performed to generate the first offspring generation *Q*_0_ (*N* = 200). To compile the next parent generation *P*_1_, the individuals of *P*_0_ and *Q*_0_ were combined and the individuals were sorted according to non-domination (Pareto front 1 … *F*) and within each front according to the crowding distance (favors individuals that have a large distance to their neighbors, see [24] for details). Finally, the top N individuals were chosen to become *P*_1_, which ensured elitism.

This procedure was repeated for *G* generations, where *G* is a number determined at runtime, at which the solutions on the first Pareto front do not change for 200 generations in feature space.

### Biomarker discovery workflow

In order to detect multiple signatures based on the 19 NSCLC samples, the phosphorylation sites were first pre-filtered for missing data, i.e. only class-I sites with at least 2/3 of ratios present in each group (responder and non-responder) were considered for further analyses. Next, the 100 sites that discriminated best between responders and non-responders according to the MeanRank [31] test were selected, while ensuring that the mean difference of the features between the two groups was at least 4-fold and only one phosphosite per protein was included. This pre-selection is necessary to reduce the complexity of the subsequent Pareto optimization. The 100 top-ranking features were subjected to the NSGA-II algorithm, which aims at detecting Pareto-optimal solutions based on the three objective functions (size, separation, relevance).

After convergence, the results were filtered for solutions that were located on the first Pareto front. Since many of them were very similar and only differ in very few features, hierarchical clustering with Ward’s method was applied on the binary solution vectors to detect clusters of similar features. Subsequently, the solutions that had the smallest Euclidean distance to the cluster centroids were taken as final Pareto signatures. If more than one solution had the smallest distance, the one with the better separation score was preferred.

### Biomarker validation

For each Pareto signature, a support vector machine with linear kernel and cost parameter *C* = 1 was trained. These SVMs are the final predictors and can be used to predict new samples. To validate the signatures, we used phosphorylation site data of the six breast cancer samples. Prior to prediction, missing values were imputed by the mean of the training data class means (for details see [9]). Subsequently, the responsiveness of the six samples was predicted with each of the final predictors.

## Supporting Information

S1 Fig Example of a Pareto front in a minimization problem. The plot shows different solutions of a toy example. Blue points are feasible solutions, where those that are not dominated by any other solution are referred to as Pareto points (dark-blue). Together they form the Pareto front. The points in the lower left area represent solutions that are desired but not feasible (yellow/red).
(TIFF)
