# Supplementary figures and images for: Pareto Optimization Identifies Diverse Set of Phosphorylation Signatures Predicting Response to Treatment with Dasatinib
Source: PLoS One. 2015 Jun 17;10(6):e0128542. doi: 10.1371/journal.pone.0128542 (PMC4470654; doi:10.1371/journal.pone.0128542)

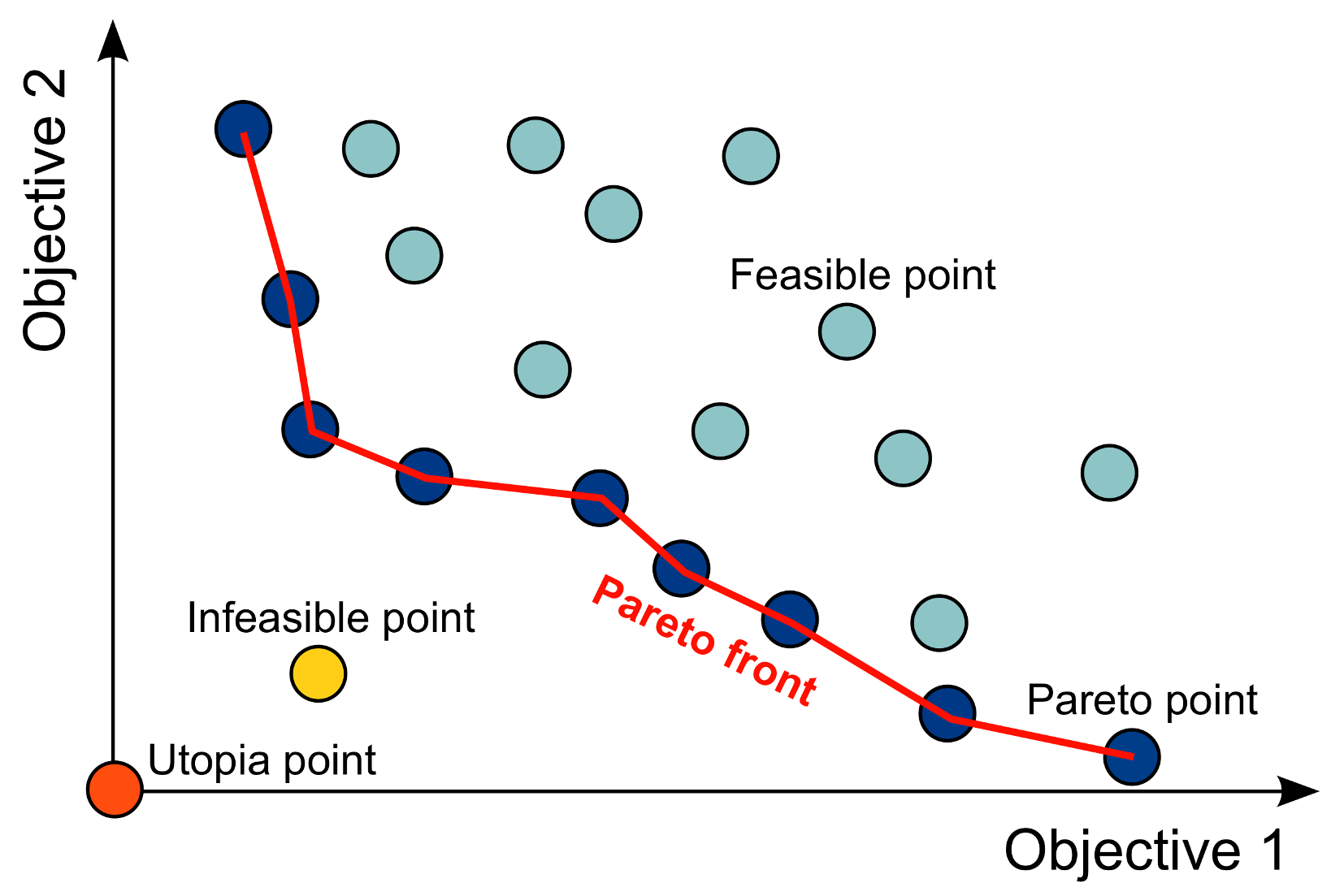

Supplement: S1 Fig — The plot shows different solutions of a toy example. Blue points are feasible solutions, where those that are not dominated by any other solution are referred to as Pareto points (dark-blue). Together they form the Pareto front. The points in the lower left area represent solutions that are desired but not feasible (yellow/red). (TIFF) [file pone.0128542.s001.tiff]
